# PSAML: A Methodological Approach for Noninvasive Computerized Hydration Level Estimation

**DOI:** 10.3390/s26113362

**Published:** 2026-05-26

**Authors:** Xin Liu, Xuezhao Kang, Liqun He, Jianrui Zhang, Huyan Ting, Xiaojun Yu

**Affiliations:** 1 School of Intelligent Manufacturing, Longdong University, Qingyang 745000, China; 2 School of Automation, Northwestern Polytechnical University, Xi’an 710072, China; zy2503513@buaa.edu.cn (X.K.); xjyu@nwpu.edu.cn (X.Y.); 3 School of Mathematics and Information Engineering, Longdong University, Qingyang 745000, China; zhangjr@ldxy.edu.cn; 4 School of Life Science and Technology, Northwestern Polytechnical University, Xi’an 710072, China; huyanting@nwpu.edu.cn

**Keywords:** hydration level detection, principal component analysis, successive decomposition index, support vector machine, classification, linear discriminant analysis, feature extraction, posture

## Abstract

Hydration level (HL) is a critical physiological indicator of human health and functional status, and accurate HL monitoring is essential for applications in healthcare, sports, and wellness assessment. However, existing methods are either invasive and inconvenient or noninvasive but limited by system complexity and insufficient accuracy. To address these limitations, this study proposes a methodological approach for noninvasive computerized HL estimation based on galvanic skin response (GSR) signals, termed the PSAML approach, which integrates principal component analysis (PCA), successive decomposition index (SDI), and machine learning (ML) classifiers. A representative GSR dataset was collected from three healthy subjects under dehydrated, normal, and overhydrated states in sitting, standing, and posture-independent scenarios. After preprocessing, including outlier removal, Butterworth filtering, and time-window segmentation, conventional time-domain features were extracted and compared with PCA- and SDI-based representations. Six ML algorithms were used for classification. The results show that the conventional feature method achieved a maximum accuracy of 63.97%, whereas PCA-based feature reduction significantly improved performance, with PCA+SVM, PCA+LR, and PCA+LDA achieving accuracies above 99% in most cases. SDI-based features also demonstrated strong performance with suitable classifiers under smaller time windows. These findings demonstrate that the proposed PSAML approach provides an accurate and efficient solution for wearable noninvasive HL monitoring.

## 1. Introduction

Hydration refers to the process by which the human body takes in and absorbs water. Dehydration and overhydration represent two extreme states of hydration, both of which can lead to illness and even death. Dehydration, characterized by insufficient body water, commonly results in disorders of the gastrointestinal, urinary, metabolic, circulatory, and neurological systems [1]. According to the World Health Organization, diarrheal disease is the second leading cause of death among children under five years of age, accounting for an estimated 370,000 child deaths globally in 2019. Furthermore, approximately four billion cases of diarrhea are reported annually, with dehydration being the most serious complication. Dehydration is also highly lethal in the elderly, with a mortality rate of 17% within 30 days and nearly 50% within one year [2]. Overhydration, also known as water intoxication, can trigger confusion, nausea, vomiting, and, in severe cases, seizures, coma, and even death [3]. Maintaining proper hydration levels not only prevents such adverse outcomes but is also vital for performing daily activities, as adequate hydration supports the body’s systems by providing the necessary energy and nutrients for proper functioning.

Accurate hydration level (HL) detection is essential for maintaining proper HL status. As established by bioelectrical impedance analysis (BIA), a clear correlation exists between human HL and body resistance. Body electrical resistance ranges from a few ohms to several thousand ohms depending on water content. Moreover, when an electrical current passes through the body, approximately 99% of the current flows through the skin. Therefore, optimizing algorithms for the measurement and analysis of skin conductance response (SCR) can facilitate the development of non-invasive wearable solutions for HL monitoring.

Automated monitoring and estimation of human HL is a promising technology for healthcare applications and has consequently attracted numerous studies investigating various HL monitoring methods, including those based on body temperature and galvanic skin impedance. In this research, we used an electrodermal activity (EDA) sensor to measure skin electrical resistance and obtain galvanic skin response (GSR) signals. While the GSR signal is highly sensitive to rapid, sympathetic nervous system arousal and emotional changes (known as phasic responses), it also reflects the baseline Skin Conductance Level (SCL, or tonic responses). Systemic fluid consumption significantly alters overall total body water, which is directly correlated with skin water content and baseline electrical resistance. Therefore, to reliably estimate hydration levels, our data preprocessing specifically involves filtering out high-frequency interference and applying large time-window segmentations (e.g., 30 s to 75 s). This algorithmic strategy effectively filters out the impulsive, emotion-driven phasic noises, allowing us to accurately capture the slow-changing tonic characteristics associated with systemic hydration states.

Several studies have explored time-domain feature extraction methods for galvanic skin response (GSR) signals in the context of hydration monitoring. Rizwan et al. pioneered a non-invasive machine learning-based approach for estimating human hydration status using galvanic skin response signals. Their study collected GSR data from subjects in sitting and standing postures under dehydrated and normal hydration states, extracted multiple time-domain features, and compared six ML algorithms. The results showed that the K-nearest neighbors algorithm, with specific feature combinations and time-window configurations, achieved a maximum accuracy of 87.78%, providing a groundbreaking direction for wearable, non-invasive hydration monitoring [4]. Liaqat et al. extended this approach by incorporating deep learning techniques. They collected GSR data from subjects in sitting, standing, and walking postures and classified hydration status into three finer categories: normal hydration, mild dehydration (8 h without fluid intake), and severe dehydration (16 h without fluid intake). Based on eight conventional ML algorithms and four deep learning models, they proposed a hybrid model in which a bidirectional LSTM network achieved a maximum accuracy of 97.83%, substantially improving estimation precision [5]. Similarly, Liaqat’s team and Priya K.V.’s team introduced a time-domain feature extraction method identifying six features related to skin hydration levels, including mean, standard deviation, square root, percentile, kurtosis, and skewness [6,7]. Following an approach similar to that of Ali Rizwan et al., these features were aggregated and classified using various machine learning algorithms. Priyanka Das’s team proposed a seven-feature extraction method incorporating amplitude, root mean square, mean, median, standard deviation, skewness, and kurtosis, followed by subsequent data analysis [8]. Furthermore, Değer Ayata’s team adopted a time-domain statistical feature extraction method, extracting multiple attributes from GSR signals—including minimum, maximum, mean, standard deviation, variance, skewness, kurtosis, median, zero-crossing, entropy, mean energy, moments, and signal value variation—and used them to train and test a random forest classifier [9]. While this multi-feature extraction approach preserves the core characteristics of the data, there remains substantial room for improving the accuracy achieved through feature combinations, and classification accuracy remains suboptimal. Moreover, the most effective feature combinations and machine learning algorithms often vary across different samples, which increases the effort required for feature and algorithm selection and limits generalizability.

Md. Toufick E Elahi et al. preprocessed GSR signals through normalization and filtering before temporal feature extraction [10]. They employed nonlinear parameters—including correlation dimension (CD), Lempel-Ziv complexity (LZC), Shannon entropy (SE), and the Hurst exponent—to analyze the chaotic characteristics of GSR signals under different conditions. After extracting features such as mean, standard deviation, entropy, and power spectral density, they applied the chi-square feature selection method to identify the most relevant features. This approach reduces data dimensionality, denoises the data, and facilitates interpretability of model predictions. Additionally, selecting features with the highest correlation to the target variable can improve model generalization. However, a limitation of this method is the potential for information loss, as discarding certain details from the original data may adversely affect model performance if inappropriate features are selected.

To address the aforementioned limitations, the primary objective of this study is to present a methodological approach for noninvasive computerized hydration level estimation. As a methodological study, rather than conducting a large-scale clinical trial, we designed a two-stage validation strategy to rigorously verify the proposed computational pipeline. First, we utilized a strictly controlled, self-collected preliminary dataset (three subjects) to establish the algorithm and isolate features under extreme hydration states. Second, we externally validated the framework using an independent public dataset to ensure its generalizability. By employing Principal Component Analysis (PCA) [11,12] and the Successive Decomposition Index (SDI) [13], we developed the PSAML framework as a foundational methodology based on galvanic skin response (GSR) signals.

The remainder of this paper is organized as follows: Section 2 describes the data acquisition methodology. Section 3 details the hydrological data processing pipeline, including the conventional time-domain feature extraction method, as well as the PCA and SDI methods built upon it, followed by the classification procedure. Section 4 presents the classification results of the traditional method, PCA, and SDI, and introduces the proposed PSAML approach for hydration level estimation. Section 5 analyzes the classification results from multiple perspectives and compares the PSAML approach with traditional methods. Section 5 concludes the paper with a summary of the findings.

## 2. Materials and Methods

**Figure 1:**
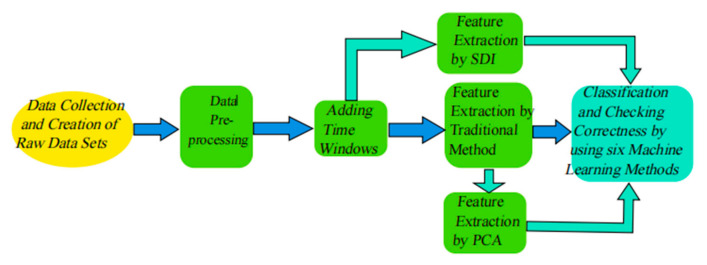
The overall diagram for electrical skin response signal processing.

The proposed non-invasive hydration estimation approach involves several key stages, from initial signal acquisition to final data classification. Once the raw electrical skin response signals were collected from the subjects, the data were processed and the signal features were extracted for classification. The overall processing steps are illustrated in Figure 1 and will be explained in detail in the following subsections.

### 2.1. Data Acquisition

#### 2.1.1. Subjects and Hydration States

Data was collected from three healthy participants with a mean age of 20 years. None of the participants had any known symptoms of edema or dehydration. The study protocol was reviewed and approved by the Institutional Review Board (IRB) at Northwestern Polytechnic University (Approval No. YX202602060). Data were labeled into three categories: overhydrated, normal, and dehydrated, following established criteria [4,5]. Data was classified as dehydrated when the participant had consumed no fluids or food for at least eight hours prior to collection. Data were classified as overhydrated when the participant had consumed a large amount of water within the past hour or had ingested water multiple times before collection. All other cases were labeled as normal.

#### 2.1.2. Hardware Setup

**Figure 2:**
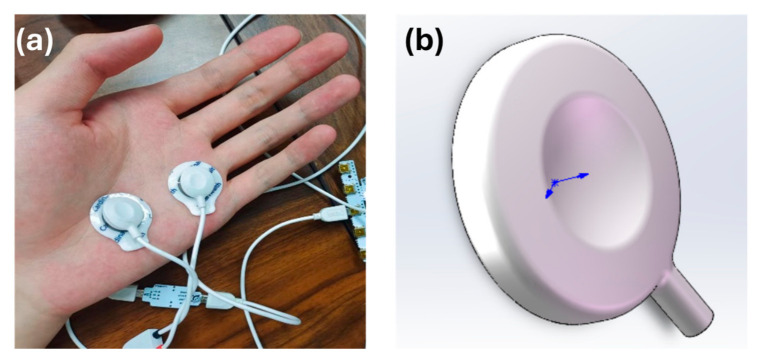
Experimental configuration: (**a**) diagram of data collection, (**b**) the 3D model of the electrode housing.

Measurements were performed using the gel electrode integrated with the EDA sensor of the BITalino Plugged Kit (BT, PLUX). Skin conductance level is quantified as the galvanic skin response (GSR) in microsiemens (µS). The electrode housing for GSR signal acquisition was 3D-printed using an FDM printer with PLA material at 100% infill to ensure structural stability and consistent skin–electrode contact. The experimental configuration is shown in Figure 2a, and the 3D model of the electrode housing is illustrated in Figure 2b. This approach offers practical value for advancing personalized and wearable hydration monitoring systems. Data were acquired at a resolution of 16 bits and a sampling rate of 1 kHz, which is the highest precision option available on the BITalino instrument [4]. The BITalino instrument is connected to a personal computer (PC) via its integrated Bluetooth module, and the signals are captured using the Opensignals software (PLUX Wireless Biosignals, Lisbon, Portugal) supplied with the instrument. The GSR of the instrument is calculated as

(1) $$GRS=\frac{1}{R}$$

where R denotes skin resistance in MΩ as below,

(2) $$R=1-\frac{C}{2^{n}}$$

#### 2.1.3. Experimental Protocol

Data were collected under two body postures: sitting and standing. For both the overhydrated and dehydrated states, data was acquired in each posture. Additionally, a posture-independent dataset was created by combining data from both postures. A total of three hours of data were collected per participant. For each hydration state, one hour of data was recorded: half an hour in the sitting posture and half an hour in the standing posture. The collection procedure involved recording half an hour of standing data, followed by a short break, and then half an hour of sitting data, completing one experimental session.

**Figure 3:**
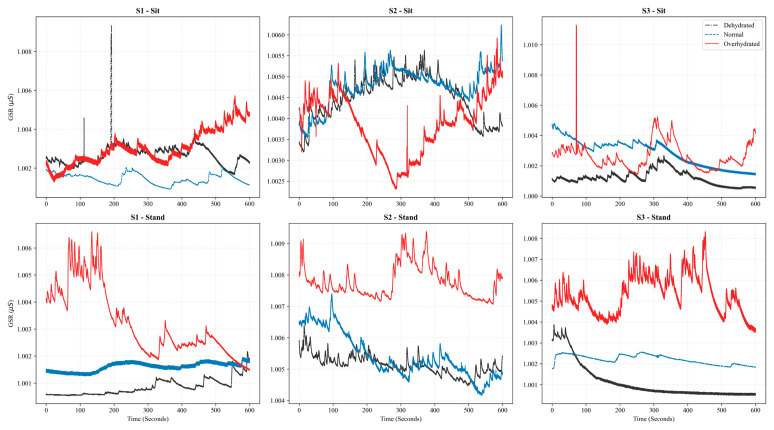
Original raw dynamic GSR records for three subjects across three hydration states.

To comprehensively address the dynamics of the acquired signals and demonstrate their universal physiological characteristics prior to processing, Figure 3 illustrates the original dynamic GSR records for all three subjects across both physical postures (sitting and standing). To authentically depict the raw signal quality without overcrowding the visualization, a representative 10 min (600 s) window is plotted for each scenario using the original 1 kHz sampling rate, completely without smoothing or downsampling.

As clearly observed across all subplots, the raw signals naturally exhibit inherent individual baseline differences and significant high-frequency fluctuations caused by spontaneous minor movements. Despite this complex physiological noise, a striking and consistent stratification in the macroscopic tonic skin conductance level emerges: the overhydrated state (red solid line) consistently maintains the highest conductance, followed by the normal state (blue dashed line), while the dehydrated state (black dashed line) reliably exhibits the lowest conductance. This uniform pattern provides robust visual evidence that increased total body water universally enhances skin electrical conductance, regardless of the subject or posture. More importantly, the highly non-stationary nature of these raw records strictly underscores the critical necessity of our subsequent preprocessing approach. It visually justifies why applying Butterworth filtering and large time-window segmentations is absolutely essential to effectively eliminate transient phasic artifacts and reliably isolate these hydration-related tonic features for the PSAML approach.

## 2.2. Data Preprocessing

First, the raw signal TXT files from the BITalino instrument were imported into MATLAB (R2024a). Outliers were subsequently identified and removed using the built-in outlier detection function. Next, filtering was performed using a Butterworth filter. While a typical GSR signal encompasses a wider frequency range that includes rapid, emotion-driven phasic responses (SCR, typically up to several Hz), this study specifically focuses on the slow-changing Skin Conductance Level (SCL). SCL is intrinsically associated with systemic hydration and primarily lies below 0.2 Hz. For the purpose of hydration estimation, higher-frequency phasic responses and movement artifacts act as interference. Therefore, a second-order Butterworth filter with a strict cutoff frequency of 0.3 Hz was intentionally applied to isolate the relevant tonic features (SCL) and eliminate high-frequency noise. Owing to the large volume of the collected signal dataset, a time window was added to the filtered data prior to time-domain feature extraction. Four time intervals were selected: 30 s, 45 s, 60 s, and 75 s. The corresponding numbers of merged windows for each interval are presented in Table 1.

## 2.3. Feature Extraction Based on Successive Decomposition Index (SDI) and Principal Component Analysis (PCA)

Principal Component Analysis (PCA) is a multivariate data analysis technique designed to reduce data dimensionality while retaining a substantial amount of information. The application of PCA in this study proceeded as follows.

First, time-domain feature extraction was performed on the data within each time window. Statistical features were calculated for each window based on its size. For example, when using a 60 s window, the data were divided into non-overlapping 60 s segments, and the nine time-domain features were computed per segment. For all test subjects, feature values for the three hydration states (dehydrated, normal, overhydrated) under various postures were calculated within each time window. Subsequently, the feature values for the three hydration states under the same posture and same time window were aggregated into a single sample. Label values were assigned as follows: dehydrated = 0, overhydrated = 1, normal = 2.

After combining data from all test subjects, each time window comprised three posture samples, yielding a total of twelve samples across the four time-windows (30 s, 45 s, 60 s, 75 s). The first nine columns of the sample matrix represent the nine time-domain statistical features—specifically, minimum, mean, variance, entropy, standard deviation, range (maximum minus minimum), median, mode, and kurtosis—following the approaches in [4,5,6,7,9]. The tenth column contains the label values.

**Figure 4:**
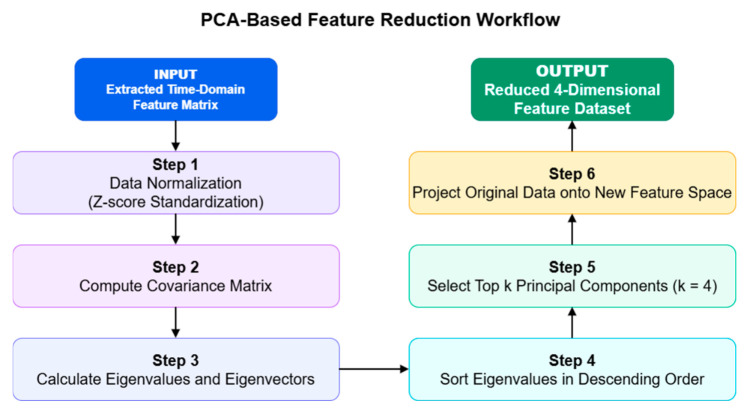
Standard flowchart of the PCA feature extraction algorithm.

Following time-domain feature extraction, dimensionality reduction was applied to the nine-dimensional feature vectors. In this study, the PCA method was configured to reduce the dimensionality to 1, 2, 3, or 4 dimensions. As shown in Figure 4, PCA was used to obtain the reduced-dimensionality data for each test subject. A mixing function was then employed to combine the reduced feature data for each hydration state across the three postures and different time windows for all subjects. The combined data were randomly shuffled using a shuffling function to ensure unbiased evaluation. Extensive experimentation revealed that the PCA method achieved the best classification accuracy when the dimensionality was reduced to 4; the results with other dimensions were marginally inferior. Therefore, for this project, the PCA method was set to reduce the dimensionality to 4.

The Successive Decomposition Index (SDI) method [13] draws inspiration from the discrete wavelet transform (DWT). In the initial stage of DWT, a time signal of length n is passed through both low-pass and high-pass filters. In subsequent layers, the output of the low-pass filter is again subjected to high-pass and low-pass filtering, and this process is repeated for a specified number of decomposition levels. The coefficients from each decomposition level are then used for feature extraction.

The initial step involves calculating the absolute mean value of the EEG signal, denoted as $S^{+}$. To obtain this value, each dataset acquired in the preceding period is treated as S, and a summation function is applied. Subsequently, the mean is computed, resulting in $S^{+}$.

(3) $$S^{+}=\frac{1}{n}\sum_{1}^{n}\left|S_{i}\right|$$

The subsequent step involves computing the mean difference, denoted as $S^{-}$ of the signal. This is determined by taking the mean of the continuous differences between non-overlapping pairs of time signals. Mathematically, it can be expressed as follows:

(4) $$s^{\left(1\right)}=\left\{\frac{s_{1}-s_{2}}{2}\right.,\frac{s_{3}-s_{4}}{2}\ldots ,\frac{s_{n-3}-s_{n-2}}{2},\left.\frac{s_{n-1}-s_{n}}{2}\right\}$$

where S^(1)^ has a length of $\frac{n}{2}$. Similarly, S^(2)^ can be calculated as

(5) $$s^{\left(2\right)}=\left\{\frac{{s_{1}}^{\left(1\right)}-{s_{2}}^{\left(1\right)}}{2}\right.,\frac{{s_{3}}^{\left(1\right)}-{s_{4}}^{\left(1\right)}}{2}\ldots ,\frac{{s^{\left(1\right)}}_{n/2-3}-{s^{\left(1\right)}}_{n/2-2}}{2},\left.\frac{{s^{\left(1\right)}}_{n/2-1}-{s^{\left(1\right)}}_{n/2}}{2}\right\}$$

The process of calculating S^(k)^ (where k is the number of iterations) continues until a coefficient is obtained, with the final coefficient representing the mean difference term S^−^. The number of iterations needed to compute S^−^ can be determined as follows: k = 3.33 log10(n), and the total number of coefficients at each step is. The next step involves computing two new terms, S^++^ and S^−−^, as follows:

(6) $$s^{++}=\frac{s^{+}+s^{-}}{2}$$

(7) $$s^{--}=\frac{s^{+}-s^{-}}{2}$$

The terms S^++^ and S^−−^ establish the relationship between S^+^ and S^−^. Furthermore, these four coefficients together constitute a square matrix denoted as Z.

Initially, the iteration coefficient k is determined using the formula k = 3.33 log10(n). Subsequently, the process iterates k times, with S^k−1^ being updated to S^k^ at each iteration, until a single value, S^−^, remains after the KTH iteration. To find the Z matrix, S+ and the provided formula are utilized.

(8) $$SDI=\log_{10}\left(\frac{n}{k}\left(S^{+}S^{++}-S^{-}S^{--}\right)\right)$$

After computing the product of matrix Z and the scalar factor nk, the base-10 logarithm of the result is taken. The SDI is then calculated for each dataset individually, producing results for all experimental cases.

**Figure 5:**
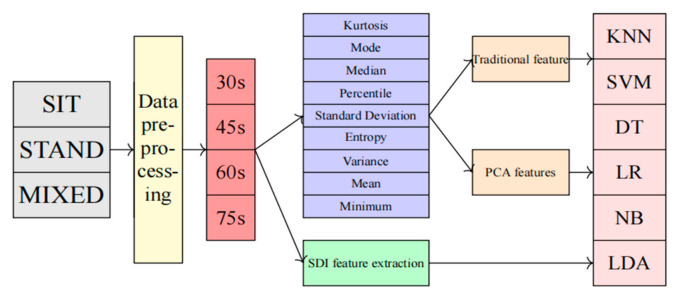
Data processing flow diagram.

The feature data derived from the SDI method is one-dimensional. Following the same procedure used for PCA, a mixing function was applied to combine the SDI feature data for each hydration state across the three postures and different time windows for all test subjects. The combined data were then randomly shuffled using a shuffling function. Finally, the characteristic values for each hydration state under the three postures and different time windows were obtained for the SDI method. The overall data processing workflow is illustrated in Figure 5.

## 2.4. Classification Algorithms

The data classification process is conducted as follows: six machine learning (ML) algorithms [4,5,6], namely, Plain Bayes (NB), Support Vector Machine (SVM), Decision Tree (DT), K Nearest Neighbor Algorithm (KNN), Linear Discriminant Analysis (LDA), and Logistic Regression (LR), were used in this study, and the hyperparameters for all six algorithms are shown in Table 2.

All the algorithm and feature combinations were evaluated separately for each time-window size and posture. For the K-nearest neighbors (KNN) algorithm, the optimal K-value (ranging from 1 to 20) was determined by traversing all integers in this range and comparing the average correctness across tests. In total, a×b×c combinations were evaluated to identify the optimal configuration, where a represents the number of time windows, b the number of subject postures, and c the number of algorithms.

For each model, 70% of the feature set data was used for training, while the remaining 30% was held out as an independent test set for final correctness evaluation. Correctness is defined as the ratio of the number of correct predictions to the total number of actual instances. All correctness values reported in this study represent the average correctness obtained by running the same model 1000 times under identical conditions.

## 3. Experimental Results

### 3.1. Classification Results Using Conventional Time-Domain Features

The nine conventional time-domain features—minimum, mean, variance, entropy, standard deviation, range (maximum minus minimum), median, mode, and kurtosis—were calculated for the three hydration states (dehydrated, overhydrated, normal) across each posture and time window for the three test subjects [4,5,6,7,9]. For each combination of posture and hydration state within a given time window, the data from the three subjects were merged into a single sample for principal component analysis (PCA). Label values were assigned as follows: dehydrated = 0, overhydrated = 1, normal = 2. Within each time window, the three posture samples were combined to yield a total of twelve samples.

Analysis of the nine time-domain features revealed that the dehydrated state exhibited significantly lower values for certain parameters (e.g., minimum, mean, median, and mode) compared to both the overhydrated and normal states. The variance of the dehydrated state was slightly larger than that of the other two states. Feature values for overhydrated and normal states were of similar magnitude, with overhydrated values consistently higher.

**Figure 6:**
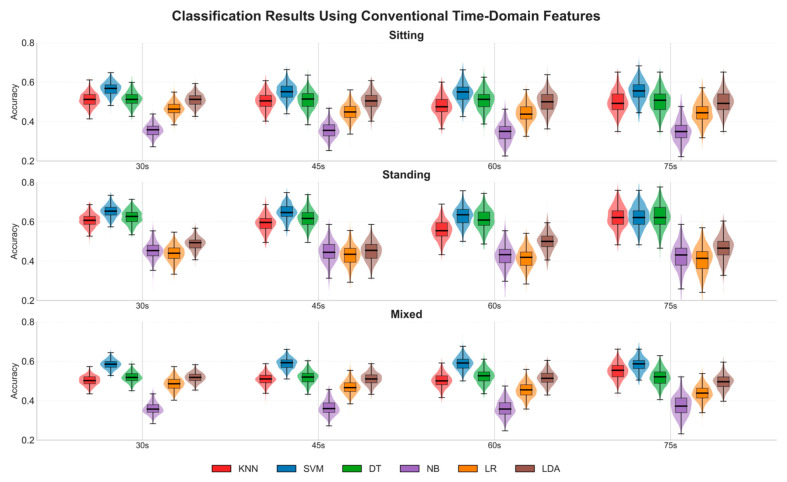
Classification results using conventional time-domain features. The violin plots illustrate the distribution, median, and variance of the accuracy across 1000 iterations for different machine learning algorithms and time windows in Sitting, Standing, and Mixed postures.

Based on these nine extracted features, six machine learning algorithms were used for classification. The experimental results are presented in Figure 6. As shown, the overall classification accuracy achieved using traditional time-domain features was low, with the highest accuracy of 63.97% obtained for the standing posture within this dataset. For comparison, Ali Rizwan et al. performed cross-combination and validation of features, achieving a maximum accuracy of 87.78% [4]. Although this represents an improvement over direct classification using all nine features, there remains considerable room for further enhancement.

### 3.2. Classification Results Using PCA-Based Feature Extraction

For each of the three test subjects, the nine time-domain features (minimum, mean, variance, entropy, standard deviation, range, median, mode, kurtosis) were first calculated for the three hydration states across all postures and time windows [4,5,6,7,9]. Principal Component Analysis (PCA) was then applied to the extracted feature set. Through extensive experimentation, it was determined that reducing the dimensionality to 4 yielded the best performance in subsequent classification tests; other reduced dimensions gave marginally inferior results. Consequently, the PCA method was configured to output 4-dimensional feature vectors for each sample.

After dimensionality reduction, the PCA-transformed features for the three hydration states under the same posture and same time window were merged into a single sample. Label values were assigned as follows: dehydrated = 0, overhydrated = 1, normal = 2. After mixing the data from the three subjects, each time-window contained three posture samples, resulting in a total of twelve samples across the four time-windows (30 s, 45 s, 60 s, 75 s).

The reduced-dimensionality data represent linear combinations of the original features, ordered by the amount of variance they explain. The feature extraction results of the PCA method (with reduction to 4 dimensions) revealed clear separation among the three hydration states. This separation was evident in the sign (positive vs. negative), absolute magnitude, and mean values of the resulting components.

**Figure 7:**
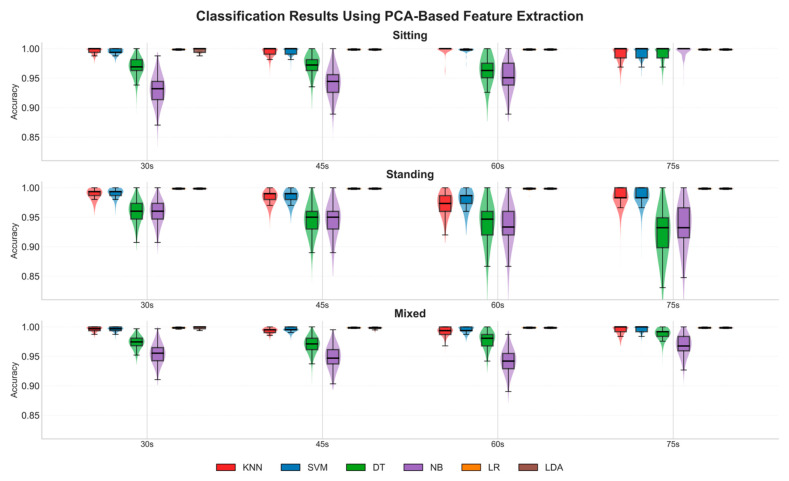
Classification results using PCA-based feature extraction. The violin plots illustrate the distribution, median, and variance of the accuracy across 1000 iterations for different machine learning algorithms and time windows in Sitting, Standing, and Mixed postures.

The experimental results are presented in Figure 7. As shown, applying PCA to reduce the data to 4 dimensions significantly improved classification accuracy across all four time-windows. Among the evaluated classifier combinations, “PCA+LR”, “PCA+LDA”, and “PCA+SVM” achieved the best performance, with correctness rates consistently exceeding 99% for most samples.

This substantial improvement over traditional methods [4,5,6] can be attributed to the ability of PCA to preserve the main features of the GSR data while filtering out posture-related variations. Although body posture during data acquisition affects the raw GSR signal, the PCA transformation effectively mitigates these confounding effects, leading to higher and more robust classification accuracy.

### 3.3. Classification Results Using SDI-Based Feature Extraction

For each time window, the SDI eigenvalues corresponding to the three hydration states (dehydrated, overhydrated, normal) under the same posture were combined into a single sample. Label values were assigned as follows: dehydrated = 0, overhydrated = 1, normal = 2. After mixing the data from the three test subjects, each time window contained three posture samples, yielding a total of twelve samples across the four time windows (30 s, 45 s, 60 s, 75 s).

The SDI feature data are one-dimensional. Although the absolute differences among the three hydration states are not large, a consistent pattern emerges: the overhydrated state exhibits the highest SDI values, followed by the normal state, while the dehydrated state shows the lowest SDI values.

**Figure 8:**
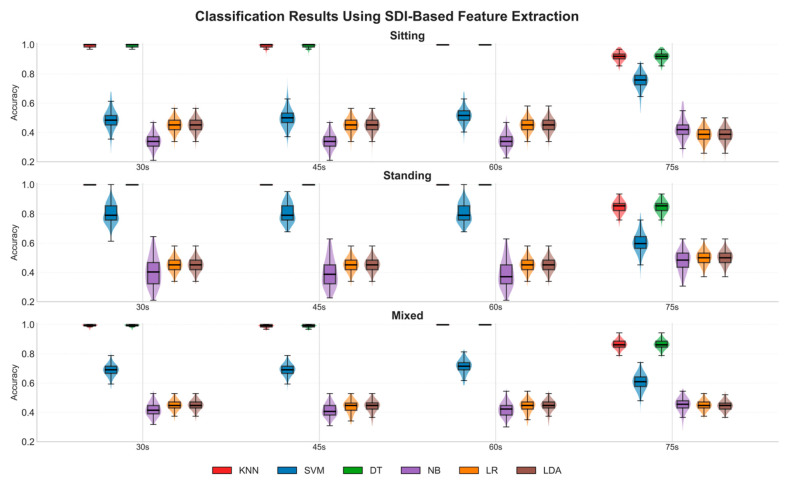
Classification results using SDI-based feature extraction. The violin plots illustrate the distribution, median, and variance of the accuracy across 1000 iterations for different machine learning algorithms and time windows in Sitting, Standing, and Mixed postures.

As with the previous experiments, six machine learning algorithms were used for classification. The experimental results are presented in Figure 8. It can be observed that the SDI method significantly improves classification accuracy for certain combinations, particularly within smaller time windows (30 s, 45 s, and 60 s). Specifically, the combinations “SDI+KNN” and “SDI+DT” achieved accuracy exceeding 99% under these window sizes.

In contrast, the combinations “SDI+SVM”, “SDI+LR”, “SDI+NB”, and “SDI+LDA” did not show substantial improvement over traditional methods; in some cases, their performance was even worse [4,5,6]. These results indicate that the SDI method is effective only when paired with appropriate machine learning algorithms. This limitation may be attributed to the one-dimensional nature of the SDI features, which influences the classification behavior of certain algorithms. Additionally, the time-window size plays a critical role in classification performance.

Based on these findings, we recommend using smaller time windows for feature extraction and selecting suitable classifiers—specifically, the “SDI+KNN” and “SDI+DT” combinations—when applying the SDI method.

Based on the experimental results from both PCA and SDI methods, we propose a new approach for non-invasive computerized hydration level estimation, termed the PSAML approach (PCA, SDI, and Machine Learning). This approach integrates three core components: Principal Component Analysis (PCA), the Successive Decomposition Index (SDI) method, and a specific machine learning algorithm. For practical hydration level detection, the appropriate combination of these components can be selected adaptively according to the chosen time window, balancing accuracy and computational efficiency.

### 3.4. Performance Comparison Across Time Windows and Classifiers

#### 3.4.1. Effect of Time Window on Classification Accuracy

To evaluate the effect of time-window size on classification accuracy, we compared the results obtained using PCA and SDI with those from conventional time-domain feature extraction methods across different window sizes. The results for sitting, standing, and all postures (posture-independent) are presented in Table 3. It should be noted that all values reported in the table represent the maximum classification accuracy achieved among six machine learning algorithms, evaluated under four time-window sizes and three feature extraction methods for the sitting posture in this experiment.

As shown in Table 3, the time-window size has little influence on the classification accuracy of traditional time-domain features and the PCA method. For the SDI method, small time windows also have a negligible effect; however, when the time window becomes relatively large (e.g., 75 s), the classification accuracy decreases noticeably.

#### 3.4.2. Effect of Classifiers on Classification Accuracy

We further evaluate the influences of different classifiers on the classification accuracy by comparing the results obtained from both PCA and SDI to those obtained from the different classifiers with the PSAML approach. The results for sitting, standing, and any postures are shown in Table 4. It is worth noting that all data shown in the table are the maximum accuracy test results of each classifier in the four time-windows under the corresponding method in this experiment.

As shown in Table 4, applying PCA for dimensionality reduction prior to correctness testing significantly improves feature data quality across all four time-windows compared to traditional methods [4,5,6]. The maximum correctness achieved in any time window exceeds 95%, representing a substantial improvement over the previous results. Notably, certain PCA-based combinations, namely “PCA+SVM”, “PCA+LDA”, and “PCA+LR”, consistently achieve correctness rates above 99%, which is a key finding of this study. In contrast, the SDI method does not consistently outperform traditional methods across all combinations. It yields good results only in specific configurations, such as “SDI+KNN” for the 30 s, 45 s, and 60 s time-windows, and “SDI+DT” for the same windows.

### 3.5. Approach Validation via Public Dataset

To address potential concerns regarding the small sample size and to rigorously verify the generalizability of the proposed PSAML approach, we conducted supplementary experiments using an independent, publicly available dataset: the shimmer-raw-dataset-dehydration. This dataset comprises physiological signals collected from 11 healthy adult participants (9 females, 2 males) using a Shimmer3 GSR+ wearable device, specifically designed for non-invasive dehydration monitoring research. The data labels were defined based on the participants’ actual fasting and hydration states: ‘dehydration’ (labeled as 1) corresponded to physiological data recorded after prolonged periods of no food or water intake (up to 15 h for some participants), while ‘hydration’ (labeled as 0) represented data collected after sufficient water consumption prior to testing. Following a standardized data preprocessing pipeline—including outlier repair, filtering, temporal windowing, and feature extraction—the 3386 min of raw data yielded a substantial 19,067 samples. Critically, this initial dataset exhibited a severe class imbalance, with 16,915 ‘hydration’ samples versus only 2152 ‘dehydration’ samples, representing an approximate 8:1 ratio. Due to this severe data imbalance, we employed an undersampling strategy on the ‘hydration’ data to ensure the classification models would not be biased towards the majority class and to achieve an equally represented, balanced dataset for training and evaluation. This balanced dataset then served as a robust platform to rigorously assess the PSAML approach’s performance [14].

**Figure 9:**
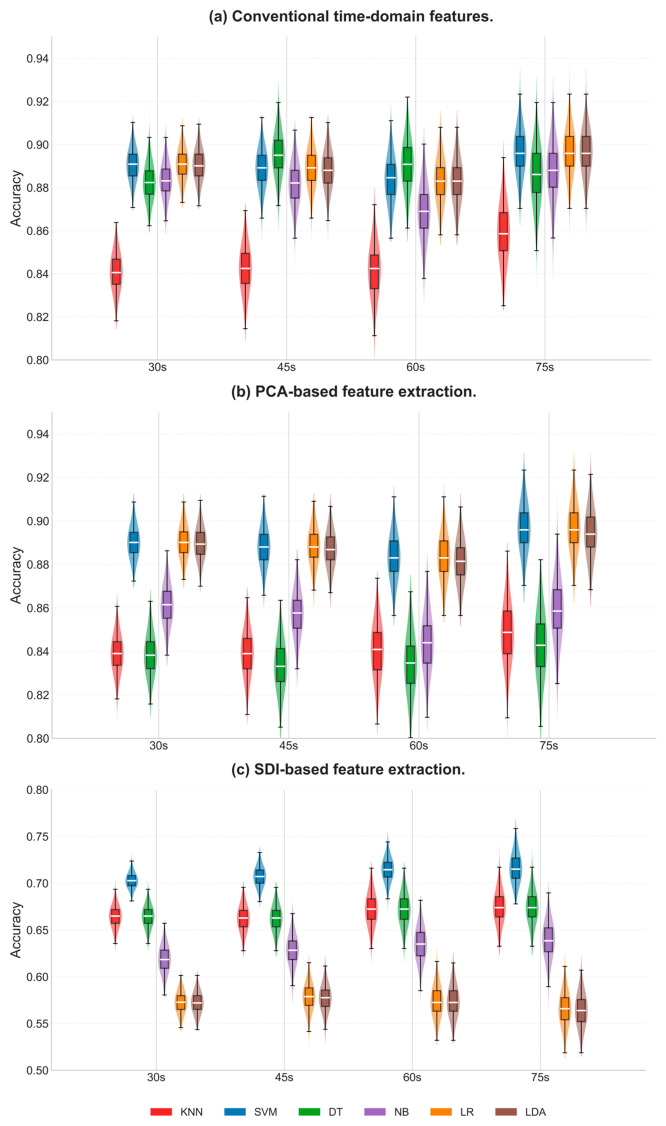
Classification results on the external public dataset.

We applied our exact analytical pipeline to this external dataset. The classification results utilizing conventional temporal features, SDI-based features, and PCA-based features across different time windows are presented via violin plots in Figure 9.

The results demonstrate that the PSAML approach maintains robust performance on unseen external data. Notably, the combinations of PCA + SVM, PCA + LR, and PCA + LDA consistently achieved high accuracies (approximately 90%) across various time windows. While the SDI method showed slightly lower overall accuracy on this specific dataset compared to our private dataset, the SVM classifier still extracted meaningful discriminative patterns. Ultimately, this external validation confirms that the high accuracy of the PCA-based feature reduction in our approach is reliable, generalizable, and not a result of overfitting to a small local cohort.

## 4. Analysis and Discussion

### 4.1. Comparison with Existing Literature

To validate the superiority of the proposed PSAML approach, we compared our results with state-of-the-art studies in non-invasive hydration monitoring. For instance, Rizwan et al. [4] utilized conventional time-domain features with a K-NN classifier, achieving a maximum accuracy of 87.78%. Liaqat et al. [5] further improved this to 97.83% using a bidirectional LSTM network, which heavily increases computational complexity. In contrast, our PSAML approach, specifically utilizing PCA-based dimensionality reduction combined with SVM, LR, or LDA, consistently achieved testing accuracies exceeding 99% across multiple time windows (as shown in the Section 3). This demonstrates that our method not only significantly reduces data dimensionality and computational overhead but also effectively filters out posture-induced noise, offering a more robust performance than traditional multi-feature methods.

### 4.2. Physiological Link from GSR to the Mechanisms of Edema

While this study computationally classifies hydration states, these findings have direct implications for understanding the physiological mechanisms of edema. Edema occurs when excessive fluid accumulates in the body’s tissues, typically in the extracellular and interstitial spaces—a state directly correlating with our “overhydrated” classification. Physiologically, an increase in interstitial fluid volume alters the bioelectrical impedance of the skin. As the skin water content rises during the onset of edema, the pathways for electrical conduction expand, leading to a measurable increase in the tonic Skin Conductance Level (SCL) captured by the GSR sensor. Therefore, the algorithm’s ability to distinctively isolate the overhydrated state from normal and dehydrated states indicates that tonic GSR monitoring could serve as an early, non-invasive early-warning mechanism for detecting fluid retention before clinically severe edema develops [15].

### 4.3. Complexity Comparison Between “PSAML” Approach and Traditional Methods

A combined analysis of Figure 6, Figure 7 and Figure 8 and Table 3 and Table 4 allows for a qualitative comparison of the three methods. When using PCA or SDI, the subject’s pose during data acquisition has little effect on the classification results. In contrast, traditional time-domain feature methods are more sensitive to postures, and classification accuracy in the standing pose is superior to that in other poses. For traditional methods, the highest correctness achieved is only 63.97%, and overall correctness remains low. Although feature extraction is relatively simple, these methods require careful selection of pose-appropriate samples and the use of parallel heuristic algorithms for feature combination, which increases the number of cases and substantially raises computational complexity.

The PCA method achieves a maximum correctness of 100% and significantly improves overall correctness. It is also simpler overall and incurs lower computational cost. The SDI method also reaches 100% correctness for some samples and requires less computation for feature extraction. However, SDI yields high correctness only when paired with appropriate machine learning algorithms, necessitating algorithm selection and thus increasing overall computational effort.

In summary, traditional methods require specific pose samples and suitable ML algorithms to achieve high correctness, leading to the greatest computational complexity and the lowest overall correctness. The SDI method requires fewer samples and thus less feature extraction computation, but its high correctness depends on appropriate ML algorithm selection, which adds computational overhead. The PCA method is the most desirable feature extraction approach, offering the highest overall correctness. It requires fewer samples, simplifies feature extraction, and minimizes computational effort by avoiding extensive ML algorithm selection.

### 4.4. Physiological Information on 9 Features with SDI Feature Information

The Skin Conductance Index (SDI) is a single-valued biomarker derived from a Galvanic Skin Response (GSR) signal of length n. It continuously captures temporal variations in the GSR signal and condenses them into a single representative value. Unlike wavelet-based or signal decomposition methods, SDI does not require selecting a suitable mother wavelet or defining the number of decomposition levels. Moreover, its computation is linear and non-complex [13].

In contrast, the nine conventional time-domain features respond only to the temporal characteristics of the GSR signal and fail to capture its frequency-domain properties. This limitation restricts their analytical capability and partly explains why traditional methods yield low classification accuracy.

### 4.5. Limitations and Future Work

Although the preliminary findings are highly promising, we acknowledge certain limitations. The dataset was collected from three subjects, which serves well for a proof-of-concept of the computational algorithm but is limited for broad clinical generalization. To mitigate this, we validated our approach using data augmentation and external public datasets (as detailed in Section 3). Furthermore, our classification relied on strict fluid-intake protocols rather than clinical urine specific gravity or blood osmolality. Future research will focus on expanding the cohort size, incorporating physiological gold standards, and exploring deep learning models to further refine the detection of intermediate, transitional hydration states.

Additionally, while a high sampling rate of 1 kHz was utilized in this proof-of-concept study to ensure maximum raw data fidelity and completely prevent aliasing, future implementations of the PSAML approach in commercial wearables will reduce the hardware sampling rate. This optimization will significantly decrease power consumption and data storage requirements without sacrificing the core slow-changing physiological features inherent to GSR.

Furthermore, we candidly recognize that the exceptional classification accuracies (e.g., reaching 100% in certain configurations) reported in this study are largely attributed to the small sample size and the use of maximally distinct, extreme hydration states. These results are not intended to represent real-world clinical accuracy but rather to validate the foundational algorithmic feasibility of the proposed approach. The true practical significance of this study lies in proposing the PSAML architecture itself: we demonstrated that combining PCA or SDI with specific ML classifiers can effectively decouple hydration-related tonic features from highly noisy, posture-affected wearable GSR signals. Validating this approach’s ability to easily distinguish extreme states serves as a crucial first step. Future research will deploy this exact approach on a larger, more diverse cohort with intermediate, subtle hydration states to evaluate its performance in complex, real-world scenarios.

## 5. Conclusions

In summary, this paper establishes a robust methodological approach for the non-invasive detection of human hydration levels. By comparing the accuracy of the principal component analysis (PCA) method (dimensionality reduced to 4) and skin dielectric impedance (SDI) method with traditional time-domain feature extraction methods, we developed a novel non-invasive computational hydration estimation approach, the PSAML approach, consisting of PCA, SDI, and specific machine learning (ML) algorithms. To advance robust hydration level estimation, this study proposes a novel feature extraction algorithm and introduces the PSAML approach, supported by a newly constructed multi-pose, multi-state GSR dataset. By significantly reducing data dimensionality, the proposed approach effectively improves detection accuracy while substantially decreasing computational costs. The effectiveness and reliability of this methodological approach were rigorously verified through a two-stage strategy: initial proof-of-concept testing on a strictly controlled multi-pose GSR dataset (three subjects), followed by successful external validation on an independent public dataset. The experimental results show that PCA-based feature processing within the PSAML approach significantly improves testing accuracy (over 90%), with PCA+SVM, PCA+LR, and PCA+LDA achieving optimal performance (≥99% accuracy). The SDI+DT and SDI+KNN combinations perform well, especially under smaller time windows. Considering posture, time window division, classifier selection, computational complexity, and practical applicability, key conclusions are drawn: For small time windows (30 s, 45 s, 60 s), five PSAML combinations (PCA+SVM, PCA+LR, PCA+LDA, SDI+DT, SDI+KNN) are recommended. For large time windows (e.g., 75 s), PCA-based combinations (PCA+SVM, PCA+LR, PCA+LDA) are preferred. Future work will investigate Neighborhood Component Analysis (NCA) for enhanced feature reduction and real-time applications.

## Figures and Tables

**Table 1:** Number of merged windows for different time windows.

Posture	State	Number of ALL Samples for Window Size			
		30 s	45 s	60 s	75 s
Stand	H	173	115	86	68
	D	165	108	80	63
	Normal	161	106	79	62
SIT	H	188	125	93	74
	D	175	116	86	68
	Normal	174	115	87	68
Independent	H	361	240	179	142
	D	340	224	158	132
	Normal	335	221	166	130

**Table 2:** Six machine learning algorithms and hyperparameter selection.

Algorithm	Hyperparameter
KNN	Metrics: [Minkowski, Euclidean, Manhattan]
	Weights: [Uniform, Distance]
	K: x, 1 ≤ x ≤ 20
SVM	Kernel: [Linear, Rbf, Poly]
	C: [0.1, 1, 10, 100, 1000]
DT	Criterion: [Gini, Entropy]
	Maxdepth: x, 3 ≤ x ≤ 25
LR	Maxdepth: x, 3 ≤ x ≤ 25
	C: [0.001, 0.01, 0.1, 1, 10, 100]
NB	NB does not involve hyperparameters.
LDA	LDA does not involve hyperparameters.

**Table 3:** Comparison of results under different time windows in sitting position.

Different Postures	Time Windows	Conventional Time Domain Feature Extraction	PCA	SDI
Sitting	30 s	51.3%	100.0%	99.48%
	45 s	50.7%	100.0%	99.01%
	60 s	50.9%	100.0%	100.0%
	75 s	49.9%	100.0%	92.26%
Standing	30 s	62.39%	100.0%	100.0%
	45 s	63.37%	100.0%	100.0%
	60 s	63.71%	100.0%	100.0%
	75 s	63.97%	100.0%	84.81%
Any pose	30 s	52.35%	100.0%	99.36%
	45 s	53.07%	100.0%	99.02%
	60 s	52.11%	100.0%	100.0%
	75 s	53.28%	100.0%	86.64%

**Table 4:** Comparison of “PSAML” approach with traditional methods for different postures.

Different Postures	Classifiers	Conventional Time Domain Feature Extraction	PCA	SDI
Sitting	KNN	49.8%	97.37%	100.0%
	SVM	49.8%	100.0%	42.91%
	DT	51.3%	99.20%	100.0%
	LR	34. 1%	100.0%	39.78%
	NB	35. 1%	99.64%	39.45%
	LDA	50.3%	100.0%	40.52%
Standing	KNN	63.97%	96.40%	100.0%
	SVM	48.24%	100.0%	49.22%
	DT	62.09%	96.00%	100.0%
	LR	31.29%	100.0%	46. 18%
	NB	44.01%	95.66%	46.38%
	LDA	49.00%	100.0%	49.22%
Any poses	KNN	53.28%	97.82%	100.0%
	SVM	51.95%	100.0%	45.63%
	DT	52.11%	98.83%	100.0%
	LR	33.31%	100.0%	45.31%
	NB	35.99%	96.88%	43.44%
	LDA	51.95%	100.0%	45.62%

## Data Availability

Data used in this study could be made available upon request.

## References

[1] Cotter J.D., Thornton S.N., Lee J.K.W., Laursen P.B. (2014). Are we being drowned in hydration advice? Thirsty for more?. Extrem. Physiol. Med..

[2] Picetti D., Foster S. (2017). Hydration health literacy in the elderly. Nutr. Healthy Aging.

[3] Farrell D.J., Bower L. (2003). Fatal water intoxication. J. Clin. Pathol..

[4] Rizwan A., Zoha A., Ozturk M., Ali A., Alomainy A., Imran M.A., Abbasi Q.H. (2020). Non-Invasive Hydration Level Estimation in Human Body Using Galvanic Skin Response. IEEE Sens. J..

[5] Liaqat S., Dashtipour K., Rizwan A., Ali A., Alomainy A., Imran M.A., Abbasi Q.H. (2022). Personalized wearable electrodermal sensing-based human skin hydration level detection for sports, health and wellbeing. Sci. Rep..

[6] Liaqat S., Dashtipour K., Arshad K., Ramzan N. (2020). Non-Invasive Skin Hydration Level Detection Using Machine Learning. Electronics.

[7] Priya K.V., Pradeep J.D. (2022). Analysis of Hydration Level Estimation Strategies using Deep Learning. Proceedings of the 2022 6th International Conference on Electronics, Communication and Aerospace Technology, Coimbatore, India, 1–3 December 2022.

[8] Das P., Das A., Tibarewala D.N., Khasnobish A. (2016). Design and development of portable galvanic skin response acquisition and analysis system. Proceedings of the 2016 International Conference on Intelligent Control Power and Instrumentation, Kolkata, India, 21–23 October 2016.

[9] Ayata D., Yaslan Y., Kamaşak M. (2016). Emotion recognition via random forest and galvanic skin response: Comparison of time based feature sets, window sizes and wavelet approaches. Proceedings of the 2016 Medical Technologies National Congress, Antalya, Turkey, 27–29 October 2016.

[10] Elahi M.T.E., Islam I.B. (2019). Galvanic Skin Response signal based Cognitive Load classification using Machine Learning classifier. Proceedings of the 2019 3rd International Conference on Electrical, Computer & Telecommunication Engineering, Rajshahi, Bangladesh, 11–13 December 2019.

[11] Sadiq M.T., Yu X., Yuan Z., Aziz M.Z., Siuly S., Ding W. (2022). A Matrix Determinant Feature Extraction Approach for Decoding Motor and Mental Imagery EEG in Subject-Specific Tasks. IEEE Trans. Cogn. Dev. Syst..

[12] Tarvainen M.P., Koistinen A.S., Valkonen-Korhonen M., Partanen J., Karjalainen P.A. (2001). Analysis of galvanic skin responses with principal components and clustering techniques. IEEE Trans. Biomed. Eng..

[13] Sadiq M.T., Yu X., Yuan Z., Aziz M.Z. (2020). Identification of Motor and Mental Imagery EEG in Two and Multiclass Subject-Dependent Tasks Using Successive Decomposition Index. Sensors.

[14] Sabry F., Eltaras T., Labda W., Hamza F., Alzoubi K., Malluhi Q. (2022). Towards on-device dehydration monitoring using machine learning from wearable device’s data. Sensors.

[15] Belabbaci N.A., Anaadumba R., Alam M.A.U. (2025). Recent advancements in wearable hydration-monitoring technologies: Scoping review of sensors, trends, and future directions. JMIR mHealth uHealth.

